# β-Sitosterol Reduces the Expression of Chemotactic Cytokine Genes in Cystic Fibrosis Bronchial Epithelial Cells

**DOI:** 10.3389/fphar.2017.00236

**Published:** 2017-05-12

**Authors:** Ilaria Lampronti, Maria C. Dechecchi, Alessandro Rimessi, Valentino Bezzerri, Elena Nicolis, Alessandra Guerrini, Massimo Tacchini, Anna Tamanini, Silvia Munari, Elisabetta D’Aversa, Alessandra Santangelo, Giuseppe Lippi, Gianni Sacchetti, Paolo Pinton, Roberto Gambari, Maddalena Agostini, Giulio Cabrini

**Affiliations:** ^1^Department of Life Sciences and Biotechnology, University of FerraraFerrara, Italy; ^2^Laboratory of Molecular Pathology, Department of Pathology and Diagnostics, University Hospital of VeronaVerona, Italy; ^3^Section of Pathology, Oncology and Experimental Biology, Laboratory for Technologies of Advanced Therapies, Department of Morphology Surgery and Experimental Medicine, University of FerraraFerrara, Italy; ^4^Italian National Health Service – USL 20 Regione Veneto and Associazione Culturale PediatriVerona, Italy

**Keywords:** *Nigella arvensis*, cystic fibrosis, inflammation, interleukin-8, cytokines, β-sitosterol

## Abstract

Extracts from *Nigella arvensis* L. seeds, which are widely used as anti-inflammatory remedies in traditional medicine of Northern Africa, were able to inhibit the expression of the pro-inflammatory neutrophil chemokine Interleukin (IL)-8 in Cystic Fibrosis (CF) bronchial epithelial IB3-1 cells exposed to the Gram-negative bacterium *Pseudomonas aeruginosa*. The chemical composition of the extracts led to the identification of three major components, β-sitosterol, stigmasterol, and campesterol, which are the most abundant phytosterols, cholesterol-like molecules, usually found in plants. β-sitosterol (BSS) was the only compound that significantly reproduced the inhibition of the *P. aeruginosa*-dependent expression of IL-8 at nanomolar concentrations. BSS was tested in CF airway epithelial CuFi-1 cells infected with *P. aeruginosa*. BSS (100 nM), showed a significant and consistent inhibitory activity on expression of the *P. aeruginosa*-stimulated expression chemokines IL-8, GRO-α GRO-β, which play a pivotal role in the recruitment of neutrophils in CF inflamed lungs. Preliminary mechanistic analysis showed that BSS partially inhibits the *P. aeruginosa*-dependent activation of Protein Kinase C isoform alpha, which is known to be involved in the transmembrane signaling activating IL-8 gene expression in bronchial epithelial cells. These data indicate BSS as a promising molecule to control excessive lung inflammation in CF patients.

## Introduction

Medicinal plants are attracting a renewed interest, since they have been a classical source of drugs for different human diseases (Newman and Cragg, 2016). This interest is particularly actual as the introduction of new drugs is now largely adopting the repurposing strategy to overcome the bottlenecks of pharmaceutical development (Strittmatter, 2014), a strategy that includes the natural medicinal products as potential repurposing source (Cragg et al., 2014). By serendipity, we focused our attention on the seeds obtained from the plant *Nigella arvensis* (*N. arvensis*), that is commonly known in native populations of Northern Africa and Asia as curative plant in traditional medicine, as its black seeds have been in use as natural remedy for over twenty centuries. The plant *Nigella* is a genre of different species, including *N. sativa*, *N. damascena*, and *N. arvensis*. Few findings were reported about extracts derived from *N. arvensis* or active principles identified in this species, while *N. sativa* was the object of different studies, reporting on different biological effects including its anti-inflammatory activities (Khader and Eckl, 2014). Regarding the possible anti-inflammatory effects of extracts derived from *Nigella* species, it has been published that *N. sativa* has therapeutic and anti-oxidant effects during lipopolysaccharide (LPS)-induced *in vivo* inflammation (Entok et al., 2014). The major biological effects of *N. sativa* are attributed to its characterized constituents, including thymoquinone, the most prominent constituent of *N. sativa* seeds. Thymoquinone is capable to reduce pro-inflammatory cytokine levels (Bai et al., 2014). In addition to thymoquinone, *Nigella* seeds contain sterols, proteins, alkaloids, saponins, and essential oils (Khader and Eckl, 2014).

Cystic Fibrosis (CF) is a severe genetic disease due to defects of the CF Transmembrane Conductance Regulator (CFTR) gene, affecting several organs. Chronic pulmonary disease is the leading cause of reduced quality and expectancy of life (Pittman et al., 2014). It is well established that chronic infection sustained by the Gram-negative bacterium *Pseudomonas aeruginosa* (*P. aeruginosa*) is a hallmark of CF lung disease, which is associated with an excessive lung inflammation characterized by huge infiltrate of neutrophils in the bronchial lumen, mainly due to the release of the neutrophil chemokine IL-8 (Bonfield et al., 1995; Khan et al., 1995; Puchelle et al., 2001; Belcher and Vij, 2010). The research regarding modern therapies to neutralize the inflammation in CF patients is aimed at finding new putative anti-inflammatory drugs displaying different mechanisms of action, in order to replace corticosteroids or ibuprofen, which posses many well-known and important side effects in addition to the great predicted benefits (Cantin et al., 2015).

The large use of the “black seeds of the desert” to mitigate the respiratory symptoms in Northern African children affected by recurrent or chronic bronchial inflammatory diseases (as anecdotal example see Supplementary Text S1), prompted us to ascertain the potential anti-inflammatory properties of these seeds. Due to the unmet need of novel anti-inflammatory drugs for chronic lung disease of CF patients, we tested the chloroform extract and the major chemical components of these seeds in CF bronchial epithelial cells, which are known to play a pivotal role in IL-8 expression and in the inflammatory response in this condition (Prandini et al., 2016). We identified *N. arvensis* as the plant originating the seeds utilized in current medicinal practice, observing that its chloroform extract is effective to reduce the expression of the key neutrophilic chemokine IL-8 in CF bronchial epithelial cells, upon exposure to *P. aeruginosa*. Among the major chemical components isolated from the seeds of *N. arvensis*, only β-sitosterol (BSS) was able to inhibit the expression of the neutrophil chemokines IL-8, GRO-α, and GRO-β, likely interfering with the protein kinase C-mediated signaling. These results indicate BSS as a promising molecule to control excessive lung inflammation in CF patients.

## Materials and Methods

### Extraction of Chemical Components from *Nigella arvensis* Seeds

Seeds of *N. arvensis* L. (Ranunculaceae) were purchased from a Berberian pharmacy in Northern Africa (Morocco). *N. arvensis* dried seeds (5.0 g) were milled in a blade grinder (0.2 mm mesh; Fritsch, Idar-Oberstein, Germany). The flour was extracted with 50 ml of hexane through ultrasound assisted maceration (20 min at 25°C), then filtered and centrifuged (3,000 rpm, 20 min). The residue was extracted with 50 ml of chloroform with the same procedure, was then re-extracted twice with 25 ml of the same solvent, for two times. All the supernatants were dried with rotary evaporator.

### Separation and Identification of Chemical Components by GC-MS Analysis

The chloroform extract was analyzed by a Varian GC-3800 gas chromatograph equipped with a Varian MS-4000 mass spectrometer (MS) using electron impact and hooked to NIST library. The column used was a Varian FactorFour VF-5ms poly-5% phenyl-95%-dimethyl-siloxane bonded phase (i.d., 0.25 mm; length, 30 m; film thickness, 0.25 μm). Operating conditions for determination of chloroform extract composition were as follows: injector temperature, 300°C; carrier (helium) flow rate, 1.5 mL/min and split ratio, 1:50. Oven temperature was increased from 230 to 320°C at a rate of 5°C/min, followed by 7 min at 320°C. The MS conditions were: ionization voltage, 70 eV; emission current, 10 mAmp; scan rate, 1 scan/s; mass range, 29–600 Da; trap temperature, 150°C, transfer line temperature, 300°C. One microliter of each sample was injected. The constituents were identified by comparing their relative retention time (KI) and the MS fragmentation patterns with pure compounds (BSS, stigmasterol, and campesterol; Sigma–Aldrich), by matching with the above mentioned mass spectra library and with those in the literature (Adams, 2007). Samples were analyzed in Gas Chromatography – Flame Ionization Detector (GC-FID) for quantitative assessment through the normalization method, without using correction factors. The relative peak areas for individual constituents were averaged on three different chromatograms. The relative percentages were determined using a ThermoQuest GC-Trace gas-chromatograph equipped with a FID detector maintained at 300°C; all the others GC conditions were the same of GC-MS method.

### Cell Cultures and Bacteria

IB3-1 cells (LGC Promochem Europe) are human bronchial epithelial cells immortalized with adeno12/SV40, derived from a CF patient with a mutant F508del/W1282X genotype. Cells were grown in the basal medium Laboratory of Human Carcinogenesis (LHC)-8 (Biofluids, Rockville, MO, USA) supplemented with 5% FBS. All culture flasks and plates were coated with a solution containing 35 μg/ml bovine collagen (BD Biosciences, Franklin Lakes, NJ, USA), 1 μg/ml BSA (Sigma–Aldrich), and 1 μg/ml human fibronectin (BD Biosciences). CuFi-1 cells, kindly donated by A. Klingelhutz, P. Karp, and J. Zabner (University of Iowa, Iowa City, IA, USA), have been derived from bronchial epithelia of a patient affected by CF (CFTR mutant genotype F508del/F508del), and were transformed by reverse transcriptase component of telomerase, hTERT, and human papillomavirus type 16 E6 and E7 gene. These cells were grown on human placental collagen type IV (Sigma–Aldrich)-coated flasks in bronchial epithelial growth medium (Cambrex Bioscience, Walkersville, MD, USA). CuFi-1 cells were also cultured into cell culture inserts (pore size of 0.4 mm) in Falcon 24-well multitrays (BD Biosciences, Franklin Lakes, NJ, USA). Cells were seeded at a density of 7 × 10^5^ cells/insert and grown in BEGM for 15 days. Transepithelial electrical resistance (TER) was measured with an epithelial voltmeter (EVOM; World Precision Instruments, Sarasota, FL, USA). The cell inserts were used for experiments when the cell monolayers reached a TER > 1000 Ω × cm^2^. The effects of N. arvensis extracts and its pure active principles (BSS, stigmasterol, and campesterol) were analyzed as elsewhere described for different chemical compounds (Nicolis et al., 2009; Borgatti et al., 2011). *P. aeruginosa*, PAO1 laboratory strain, was kindly provided by A. Prince (Columbia University, New York). Bacteria were grown in trypticase soy broth (TSB) or agar (TSA) (Difco, Detroit, MI, USA) as described (Dechecchi et al., 2008).

### Proliferation Assay

IB3-1 or CuFi-1 cells were seeded at a density of 200,000 cells in 24 well plates in LHC-8 medium in the presence of 5% FBS. After adhesion, *N. arvensis* extract was added at serial dilutions (as indicate in the figures) and incubated for further 24 h or 48 h. Cells were washed with PBS, detached with trypsin/EDTA and resuspended in DMEM medium. Finally, cells were counted with a Sysmex XE-2100 Cytometer.

### Anti-bacterial Assay

The anti-microbial activity of *N. arvensis* extracts was determined by following the procedure for the Minimum Inhibitory Concentration (MIC) of the Clinical and Laboratory Standards Institute (CLSI), former National Committee for Clinical Laboratory Standards (NCCLS). Briefly, *P. aeruginosa* (PAO1 strain) was cultured on agar plates of TSA overnight at 37°C. The range of *N. arvensis* extract concentration tested (as indicated in the figure) was prepared in 15 ml tubes containing 5 ml of TSB. A McFarland 0.5 standard concentration of *P. aeruginosa* (20 μl) was added to each tube and the samples were incubated at 37°C for 24 h. MIC is defined as the lowest concentration of compound at which there is no visible organism growth. In order to verify the absence of bacterial growth, the samples were read at 660 nm wavelength for quantitative analysis with a Beckman DU 640 spectrophotometer.

#### Adherence of PAO1 to IB3-1 Cells

PAO1 were metabolically labeled with [^35^S]-methionine according to (Saiman and Prince, 1993) with minor changes, as described (Dechecchi et al., 2008). Colonies of PAO1 from overnight TSA plates were inoculated M9 (Difco, Detroit, MI, USA) medium and grown at 37°C with shaking to a density of 10^8^ CFU/ml. 100 mCi/ml [35S] methionine (Amersham Biosciences, Uppsala, Sweden) was added to the broth and incubated at 37°C with shaking for 30 min. Bacteria were then washed twice with 10 mM NaCl and resuspended in PBS. Aliquots of bacterial suspension were plated and scintillations counted to calculate the number of bacteria associated with the counts per minute (CFU/cpm). Specific activity ranged between 40 and 1000 CFU/cpm. Metabolically labeled PAO1 were added to monolayers of IB3-1 cells and incubated at room temperature for 60 min. Unbound organisms were rinsed off the monolayers with three successive PBS washes. Cells and adherent bacteria were solubilized in 0.5 ml of 2% SDS and scintillations were counted. Specific binding was calculated by subtracting counts obtained in the presence of 100-fold excess unlabelled PAO1. Non-specific binding was about 30% of total.

### Quantitative Gene Expression Analyses by qRT-PCR

Total RNA from IB3-1 and CuFi-1 cells was purified using a High Pure RNA Isolation Kit (Roche, Mannheim, Germany), and 2.0 μg RNA were reverse transcribed to cDNA using the High Capacity cDNA Archive Kit and random primers (Applied Biosystems, Foster City, CA, USA) in a final reaction volume of 20 μl. For the Real-time qPCR, 5 μl of cDNA were used for each SYBR Green real-time PCR to quantify the relative gene expression. The cDNA (5 μl) was then amplified for 40 PCR cycles using the SYBR Green PCR Master Mix (Applied Biosystems) in a 25 μl reaction using 7900HT Fast Real-Time PCR apparatus (Applied Biosystems, Foster City, CA, USA). In order to perform the PCR reaction QuantiTect Primer assays (Qiagen, Hilden, Germany) for IL-8 (Hs_IL8_1_SG, NM_000584), GRO-α (Hs_CXCL1_1_SG, NM_001511), GRO-β (Hs_CXCL3_1_SG, NM_002090), ICAM-1 (Hs_ICAM1_1_SG, NM_000201), IL-6 (Hs_IL6_1_SG, NM_000600), TNF-α (Hs_TNF_1_SG, NM_000594), IFN-γ (Hs_IFNG_1_SG, NM_000619), IP-10 (Hs_CXCL10_1_SG, NM_001565), LPO (Hs_LPO_1_SG, NM_006151), DEFB2 (Hs_DEFB2_1_SG, NM_005218), DEFB4A (Hs_DEFB4A_1_SG, NM_004942), IL-1β (hS_IL1B_1_SG, NM_00576), GAPDH (HS_GAPDH_1_SG, NM_002046) were purchased. Changes in mRNA expression level were calculated following normalization with the GAPDH calibrator gene. Results were collected with SDS 2.3 software (Applied Biosystems), and relative quantification was performed using the Ct method. Data were analyzed with RQ Manager software 1.2 (Applied Biosystems).

### Quantitative Expression Cytokine Release

IB3-1 cells seeded on 24 wells Petri dishes were treated with vehicle alone or BSS for 16 h and then infected with PAO1 for further 4 h. Quantitative measurement of Il-8 protein release in the cell medium was measured by the Human IL-8 Instant ELISA kit (Bender MedSystems, Vienna, Austria). Cytokines released from CuFi-1 cells into tissue culture supernatants were measured by Magnetic Luminex Assay (R&D SYSTEMS, Minneapolis, MN, USA) as suggested by the manufacturer. The Luminex assay is designed for the multiplexed quantitative measurement of multiple cytokines in a single well using as little as 50 μl of sample. In our experiments, the Human Premixed Multi-Analyte Kit (R&D SYSTEMS) for the IL-8, GRO-α, GRO-β human cytokines analysis was used. 50 μl of cytokine standards or samples (supernatants recovered from treated cells) were incubated with 50 μl of anti-cytokine conjugated magnetic beads in 96-well plates for 2 h at room temperature with shaking. Plates were washed three times with 100 μl of Bio-Plex wash buffer using Bio-Plex Pro-wash Station (Bio-Rad laboratories, Hercules, CA, USA), 50 μl of diluted detection antibody were added, and plates were incubated for 1 h at room temperature with shaking. After three washes, 50 μl of streptavidin-phycoerythrin was added, and the plates were incubated for 30 min at room temperature with shaking. Finally, plates were washed three times, beads were suspended in Bio-Plex wash buffer, and samples were analyzed on a Bio-Plex 200 Array reader (Bio-Rad). Data were analyzed with Bio-Pex Manager software (Bio-Rad).

### PKCα Translocation and Microscopic Analysis

IB3-1 cells were seeded and then transfected with the Protein Kinase C isoform alpha fused to green fluorescence protein (PKCαGFP), using Lipofectamine LTX, as previously described (Chiesa et al., 2001). Microscope analysis was performed 36 h after transfection, *P. aeruginosa* strain 1:100 CFU was added to the cells, as shown in the figure. Images of PKCα translocation were recorded a different time points using a digital imaging system based on a Zeiss Axiovert 200 fluorescence microscope. The data were acquired and processed using the MetaMorph analysis program (Universal Imaging). The recruitment of the kinase is represented as plasma membrane translocation of PKCαGFP, expressed as percentage of the increase in fluorescence ratio with respect to time 0 (calculated as the ratio of plasma membrane and cytosol average intracellular fluorescence, obtained from multiple regions inside the cytosol and on the cell membrane, measured on single cell). The values are expressed as fold change vs. uninfected PAO1 control cells (0 min), referred as 100%. Indeed, the PKCα translocation was quantified by using representative line scan profile of fluorescence intensity across the cell, indicated by white diagonal lines in “0 min” and “60 min” images reported in the figure.

### Statistics

Results are expressed as mean ± standard error of the mean (SEM). Comparisons between groups were made by using Student’s *t*-test. Statistical significance was defined for ^∗^*p* < 0.05, ^∗∗^*p* < 0.01, ^∗∗∗^*p* < 0.001.

## Results

### Major Chemical Composition of *N. arvensis* Extract

**FIGURE 1:**
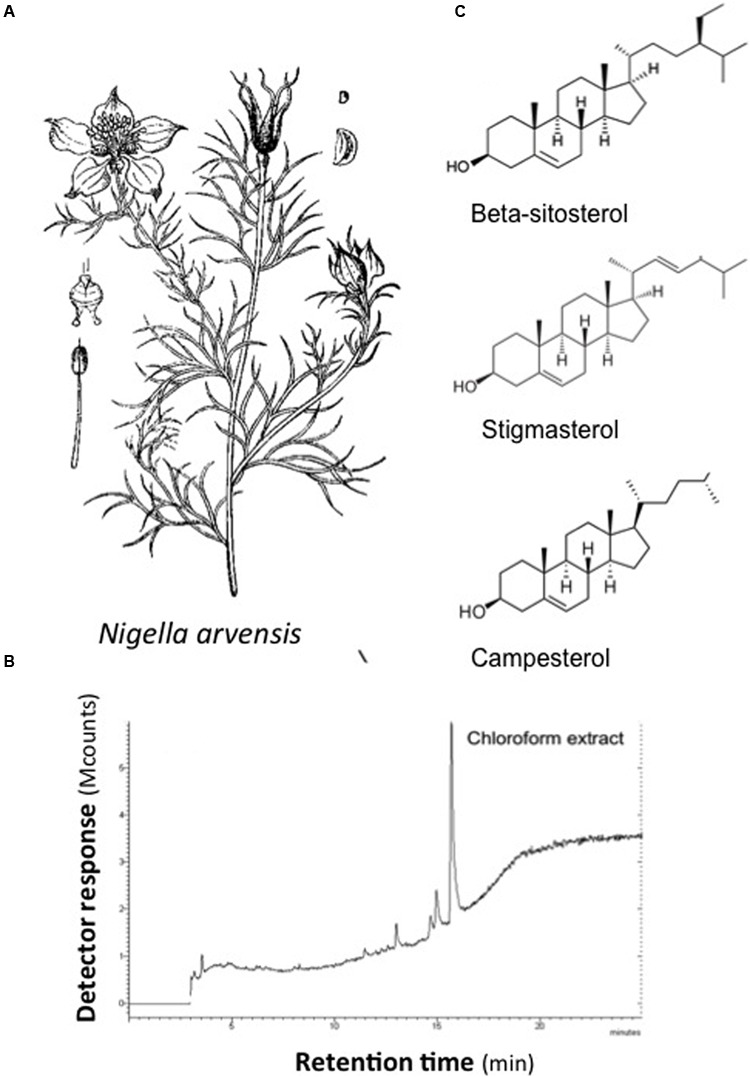
**Chemical structures of major compounds identified in extracts from *Nigella arvensis.* (A)** *N. arvensis.* **(B)** GC/MS profile of chloroform extract. **(C)** Chemical structures of β-sitosterol, stigmasterol, and campesterol.

Chloroform extract from milled *N. arvensis* seeds was analyzed. Several chemical compounds were identified as principal constituents of *N. arvensis* extracts, including BSS, stigmasterol, and campesterol (**Figure 1**). This identification procedure was performed by GC-MS analysis of chloroform extracts of *N. arvensis*. BSS, stigmasterol, and campesterol are phytosterols, cholesterol-like molecules found in plant material with the highest concentrations occurring in vegetable oils. Phytosterols act as structural components in the vegetal cell membrane, a role that is played by cholesterol in mammalian cells. The extraction yield was 0.94%. The chemical fingerprinting of *N. arvensis* extracts was achieved by GC-MS. The main phytosterols were identified by comparison between the experimental peaks and the standards molecules peaks analyzed with the same technique. The phytocomplex obtained by the chloroform extraction exhibited an important phytosterols component, constituted by stigmastan-3,5-diene (tentatively identified), campesterol, stigmasterol, and BSS, the latter being the most abundant compound, as reported in **Table 1**.

**Table 1:** Compounds identified in chloroform extract.

Compounds	Area %	rt	
Stigmastan-3,5-diene	8.53	13.00	Tentatively identified
Campesterol	2.42	14.66	Validated by the comparison with the pure standard
Stigmasterol	13.73	14.95	Validated by the comparison with the pure standard
β-Sitosterol	75.31	15.68	Validated by the comparison with the pure standard

*Compounds listed according to their elution on VF5 column.*

### Inhibition of IL-8 mRNA Expression in IB3-1 Cells Infected by *P. aeruginosa* and Treated with *N. arvensis* Extract

**FIGURE 2:**
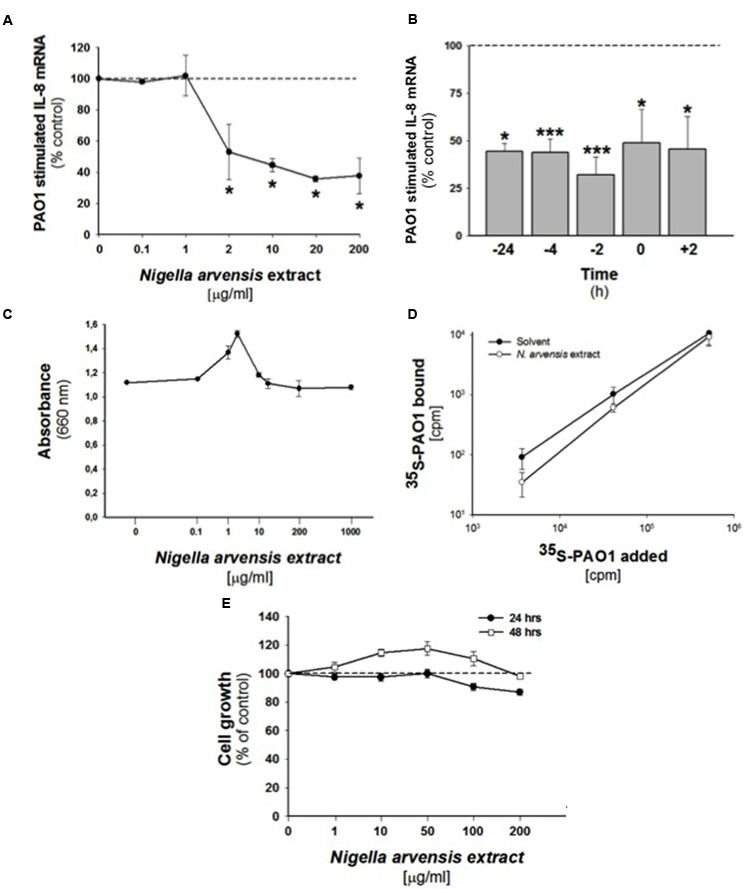
**Effect of *N. arvensis* extracts in IB3-1 cells. (A)** Effect of *N. arvensis* extracts on IL-8 mRNA expression in IB3-1 cells. IB3-1 cells were treated with the chloroform extract (solved in EtOH/DMSO 95/5) **(A)** at different concentrations (0.1–200 μg/ml) for 16 h before infection with PAO1 (100 CFU/cell) for further 4 h. IL-8 mRNA expression was quantified by qRT-PCR. Expression of IL-8 mRNA was measured by Real-Time qPCR and obtained by comparing the ratio IL-8 and the housekeeping gene GAPDH between non-infected and infected cells. The results are expressed as the % of untreated cells. Data are mean ±SEM of three independent experiments performed in duplicate. Dashed line corresponds to cells treated with solvent alone. **(B)** Effect of *N. arvensis* extract in PAO1 infected IB3-1 cells after different incubation times. The *N. arvensis* extract (10 μg/ml) was added to IB3-1 cells 24, 4, and 2 h before, simultaneously or 2 h post PAO1 infection (100 CFU/cell). IL-8 mRNA expression was measured as indicated in **(A)**. Data are mean ±SEM of three independent experiments performed in duplicate. Dashed line corresponds to cells treated with solvent alone. **(C)** PAO1 growth. Bacteria were cultured overnight at 37°C in the presence of solvent or ranging doses (0.1–1000 μg/ml) of *N. arvensis* extract. Bacterial growth was monitored by absorbance measures at 660 nm. A representative experiment performed in duplicate is shown. **(D)** Adhesion of PAO1 to IB3.1 cells. 500,000 IB3-1 cells on Petri dishes, in duplicate, were treated for 24 h with 10 μg/ml *N. arvensis* extract. Different amounts of ^35^S-PAO1, expressed as CFU/well, were added to the wells and incubated as described in section “Materials and Methods”. Data reported in the figure are the specific binding calculated by subtracting counts obtained in the presence of 100-fold excess of non-labeled PAO1 and are expressed as CFU/well. Data are mean ±SEM of three independent experiments performed in duplicate. **(E)** Effects of *N. arvensis* extracts on cell growth in IB3-1 cells. IB3-1 cells were incubated with increasing concentrations (1–200 μg/ml) of the chloroform extract (solved in EtOH/DMSO) of *N. arvensis* for 24 and 48 h. Cell viability was measured by cytometer analysis. Data are expressed as % control (solvent) and are relative to a representative experiment performed in duplicate. Dashed line corresponds to cells treated with solvent alone.

In order to study the possible anti-inflammatory effect of *N. arvensis* seeds, we firstly tested different concentrations of the extract in CF bronchial epithelial IB3-1 cells infected with the *P. aeruginosa* laboratory strain PAO1. The neutrophil chemokine IL-8 transcript was quantified. As shown in **Figure 2A**, the extract significantly inhibited, the PAO1-dependent transcription of IL-8 in IB3-1 cells by approximately 50%, starting from 2 μg/ml. The inhibitory effect of the *N. arvensis* extract was tested after different incubation times. The *N. arvensis* extract (10 μg/ml) was added to IB3-1 cells 24, 4, and 2 h before, simultaneously or 2 h post PAO1 infection (100 CFU/cell). The inhibitory effect was confirmed in all these conditions, as shown in **Figure 2B**. These findings indicate that extract of *N. arvensis* seeds reduces the inflammatory response to *P. aeruginosa* in CF bronchial cells.

### Extract from *N. arvensis* Has No Effect on Cell Growth in IB3-1 Cells

In order to exclude possible adverse effects on cell cycle, cell growth was studied in IB3-1 cells treated with the extract (1–200 μg/ml) for 24 or 48 h. No changes in cell growth were observed in IB3-1 cells treated with the *N. arvensis* extract (**Figure 2E**), also at high concentration (200 μg/ml). These data indicate that the extract of *N. arvensis* does not affect the proliferation of IB3-1 cells.

### Extract from *N. arvensis* Does Not Affect PAO1 Growth and Adherence to IB3-1 Cells

To exclude that the inhibition of *P. aeruginosa*-dependent induction of IL-8 was due to an indirect anti-bacterial effect on *P. aeruginosa*, we performed an anti-bacterial assay following the procedure for the MIC. Bacteria were cultured overnight at 37°C in the presence of solvent or ranging doses (0.1–1000 μg/ml) of *N. arvensis* extract. As shown in **Figure 2C**, no effect on bacterial growth was found, indicating that *N. arvensis* does not possess significant anti-bacterial activity on the Gram-negative bacterium *P. aeruginosa*. Considering that many pathogenic microorganisms use glycoconjugate receptors to establish contact with the host tissues (McNamara et al., 2006), the inhibition of biosynthesis of these receptors may have a major impact on the pathogenesis of infection and, consequently, on the host response induced by infection. Therefore, the anti-inflammatory effect of *N. arvensis* extract observed in IB3-1 cells could reflect the reduced expression of glycolipid receptors for PAO1. To evaluate this possibility, adherence of metabolically labeled [^35^S] methionine-PAO1 was measured in IB3-1 cells, treated or not for 24 h with the extract of *N. arvensis* (10 μg/ml). **Figure 2D** shows a dose-dependent increase of PAO1 binding to the cells, no significant differences due to the treatment with *N. arvensis* could be found. These results indicate that incubation with *N. arvensis* extract for 24 h does not affect the adherence of PAO1 to IB3-1 cells, thus suggesting that the sharp inhibition of *P. aeruginosa*-dependent IL-8 transcription after treatment with *N. arvensis* is independent from reduction of bacterial-host cell interactions.

### Extract of *N. arvensis* Reduces *P. aeruginosa* Dependent IL-8 Expression in IB3-1 Cells

**FIGURE 3:**
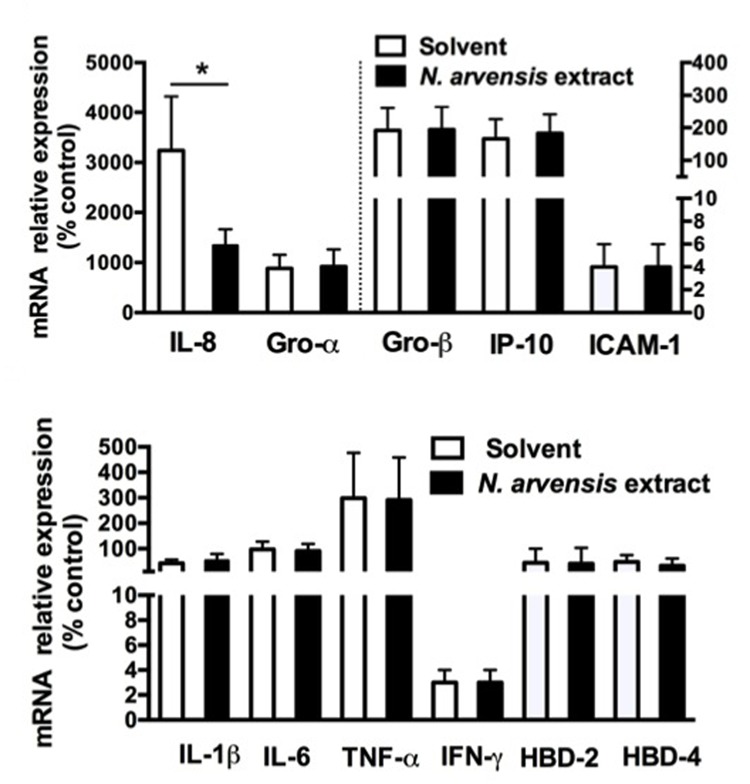
***Pseudomonas aeruginosa* modulated mRNA expression in IB3-1 cells: effect of *N. arvensis* extract.** IB3-1 cells were treated with the chloroform extract (10 μg/ml) or solvent alone 16 h before infection with PAO1 (100 CFU/cell) for further 4 h. mRNA expression was measured by real-time qPCR and obtained by comparing the ratio of target to housekeeping gene GAPDH between non-infected and infected cells.

We extended the analysis of the anti-inflammatory effect to other genes known to be involved in the host–pathogen interaction in CF bronchial epithelial cells (Dechecchi et al., 2011). As shown in **Figure 3**, infection with the *P. aeruginosa* strain PAO1 for 4 h up-modulated the expression of the major neutrophil chemokines IL-8, GRO-α, and GRO-β, of the mononuclear cells chemokine IP-10, of the adhesion molecule intercellular adhesion molecule 1 (ICAM-1) involved in leukocyte chemotaxis, of the cytokines IL-1β, IL-6, TNF-α, IFN-γ, of the antimicrobial peptides β-defensin-2 (HBD-2) and -4 (HBD-4) and of lactoperoxidase (LPO). IL-8, the most abundantly expressed cytokine in the lung of CF patients, was the only one gene to be significantly inhibited by the extract of *N. arvensis* in this cell model, as shown in **Figure 3**.

### BSS Reduces *P. aeruginosa*-Dependent Inflammatory Response in CF Bronchial Epithelial Cells

**FIGURE 4:**
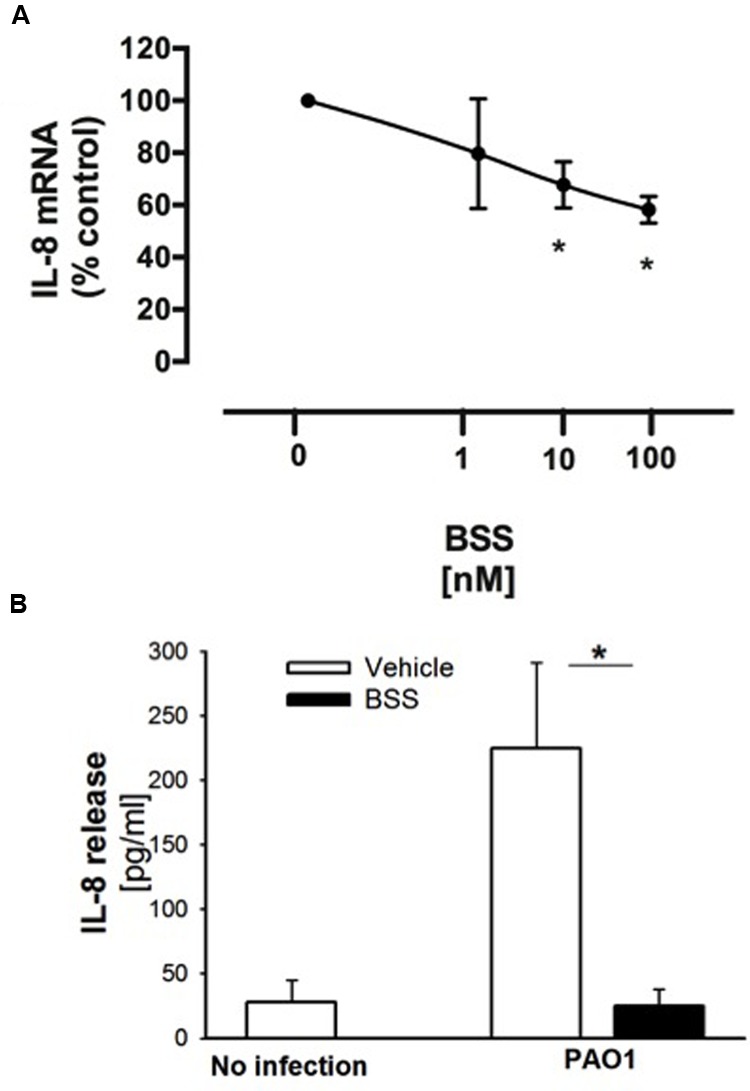
**Effect of BSS on PAO1-stimulated IL-8 expression in IB3-1 cells. (A)** IB3-1 cells were treated with ranging doses (1–100 nM) of BBS for 16 h and infected with PAO1 for further 4 h. IL-8 mRNA expression was measured as indicated in the legend of **Figure 2**. The results are expressed as the % of untreated cells. Data are mean ± SEM of three independent experiments performed in duplicate. **(B)** IB3-1 cells were treated with 100 nM BSS for 16 h and infected with PAO1 for further 4 h. IL-8 release in the supernatant was measured by ELISA assay. Representative experiment performed in duplicate.

**FIGURE 5:**
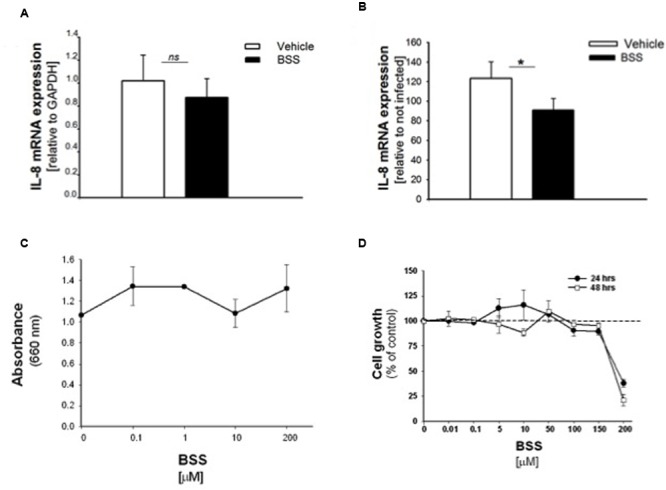
**Effect of BSS on IL-8 mRNA, bacterial growth and cell viability in CuFi-1 cells.** Cells were treated for 16 h with BSS (100 nM) and infected with PAO1 for further 4 h. IL-8 mRNA expression was measured as indicated in the legend of **Figure 2**. **(A)** Basal IL-8 mRNA expression. Data are expressed as relative to the expression of GAPDH housekeeping gene. **(B)** PAO1-stimulated mRNA expression. Data are expressed as relative to not infected cells. **(C)** PAO1 growth. Bacteria were cultured overnight at 37°C in the presence of solvent or ranging doses (0.1– 200 μM) of BSS. Bacterial growth was monitored by absorbance measures at 660 nm. A representative experiment performed in duplicate is shown. **(D)** Cell viability. CuFi-1 cells were treated with solvent alone or BSS (0.01–200 μM) for 24 and 48 h. Cell viability was recorded by cytometer analysis. Data are expressed as % control (solvent) and are relative to a representative experiment performed in duplicate. Dashed line corresponds to cells treated with solvent alone.

**FIGURE 6:**
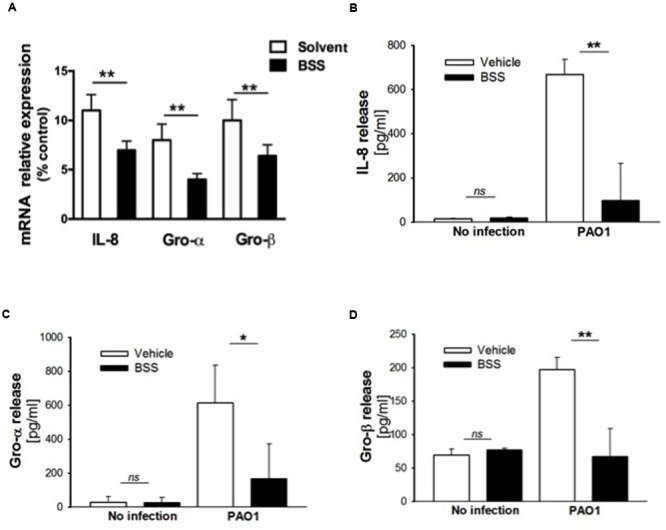
**Effect of BSS on expression of neutrophil chemokines in CuFi-1 cells. (A)** CuFi-1 cells were treated for 16 h with solvent alone or 100 nM BSS and then infected by PAO1 (10–50 CFU/cell) for 4 h. mRNA expression was measured as indicated in the legend of **Figure 2**. **(B–D)** Release of the neutrophil chemokines IL-8, Groα, and GROβ in the supernatants of CuFi-1 cells were measured by Bio-plex assay. CuFi-1 cells were treated as described for **(A)**. Representative of at least three experiments performed in duplicate.

β-sitosterol, stigmasterol, and campesterol were the three major compounds identified in the extract from the seeds of *N. arvensis*, as shown in **Figure 1**. These compounds were purchased and tested in IB3-1 cells in order to identify those reproducing the anti-inflammatory activity observed with the whole extract. The IB3-1 cells were treated with increasing doses, starting from 1 nM BSS, stigmasterol, and campesterol for 16 h before infection with PAO1. As shown in **Figure 4A**, BSS significantly inhibited the PAO1-dependent transcription of IL-8, starting from 10 nM concentration. No effect on the transcription of IL-8 was found in cells treated with stigmasterol or campesterol (data not shown). In order to check whether changes in IL-8 mRNA was directly translated into protein level, secretion of IL-8 protein was measured in cell supernatants. As shown in **Figure 4B**, BSS (100 nM) strongly inhibited IL-8 release in IB3-1 cells. The effect of BSS in IB3-1 cells was verified in CuFi-1 bronchial epithelial cells. As shown in **Figure 5**, BSS (100 nM) significantly reduces the expression of IL-8 mRNA induced by *P. aeruginosa* (**Figure 5B**). No significant effect of BSS only on IL-8 expression in basal uninfected cells was observed (**Figure 5A**). The effect of BSS on the inflammatory response to PAO1 was extended to the analysis of the other major neutrophil chemokines GRO-α, GRO-β. BSS significantly reduced the *P. aeruginosa*-dependent expression of the neutrophil chemokines IL-8, GRO-α, and GRO-β, both at transcript (**Figure 6A**) and protein levels (**Figures 6B–D**). BSS did not change the basal chemokine release.

### BSS Does Not Interfere with Bacterial Growth and Cell Viability

It has been recently reported that BSS extracted from the roots of *Caylusea abyssinica* has a moderate antibacterial activity against *P. aeruginosa*, *Staphylococcus aureus*, and *Escherichia coli* (Edilu et al., 2015). In order to verify whether the anti-inflammatory effect of BSS was due to its antibacterial property, we measured PAO1 growth in the presence of increasing concentration of BSS (from 0.01 to 200 μM). **Figure 5C** shows that BSS did not inhibit bacterial growth, at least in our experimental conditions. Therefore, We studied the cell viability of CuFi-1 cells treated with the solvent alone or increasing doses of BSS (0.01–200 μM) for 24 and 48 h. Data shown in **Figure 5D** demonstrate a cytotoxic effect of BSS only at doses higher than 150 μM.

### BSS Reduces the Activation of Protein Kinase C α Isoform Induced by *P. aeruginosa*

**FIGURE 7:**
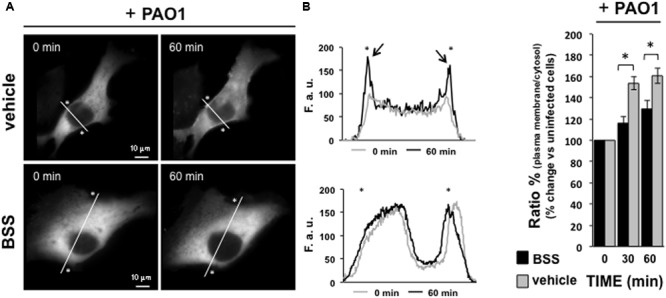
**Effect of BSS on PKCα translocation.** IB3-1 cells were seeded and then transfected with the Protein Kinase C isoform α fused to green fluorescence protein (PKCαGFP). Microscope analysis was performed 36 h after transfection, *P. aeruginosa* strain 1:100 CFU was added to the cells, as shown in the figure. **(A)** Images of PKCα translocation on the plasma membrane in the presence of *P. aeruginosa* strain PAO1 in cells pre-treated with BSS or solvent alone (vehicle). PKCα translocation was quantified by using line scan profile of fluorescence intensity across the cell, indicated by white diagonal lines in “0 min” and “60 min” images. The graphs show the comparison between the PKCαGFP fluorescence intensity profiles, expressed as fluorescent arbitrary units (F.a.u.), at 0 min and 60 min of PAO1 infection in cells pre-treated with vehicle and BSS, respectively. The asterisks indicate the edge of the cell indicative of plasma membrane. The arrows designate the enriched PKCαGFP signal at the plasma membrane in cells pre-treated with vehicle after 60 min of pathogen infection, confirming the plasma membrane translocation of PKCαGFP. **(B)** The recruitment of the kinase is expressed as percentage of the increase in fluorescence ratio with respect to time 0 of pathogen infection, calculated as the ratio of plasma membrane and cytosol average intracellular fluorescence intensity, obtained from multiple regions inside the cytosol and on the cell membrane, measured on single cell. The values are expressed as fold change vs. un-infected PAO-1 control cells (0 min), referred as 100%.

We previously observed that *P. aeruginosa* interacting with Toll-like Receptors 5 and 2 induces a pro-inflammatory cascade and activation of the intracellular Ca^2+^ pathway in which PLCB3 and PKC play key regulatory roles (Bezzerri et al., 2011b). We verified a potential effect of BSS on this latter pathway by investigating its effect on the translocation of the Ca^2+^-dependent PKCα isoform in IB3-1 cells. As shown in **Figure 7**, BSS (100 nM) significantly reduces the extent of translocation of PKCα to the plasma membrane from 30 to 60 min after *P. aeruginosa* exposure.

## Discussion and Conclusions

The urgency of finding more effective anti-infectious and anti-cancer drugs has newly opened the strategy of drug repurposing, which is aimed to discover new pharmaceutical activities for “old” clinically used drugs. A wide source of molecules for drug repurposing can be found within the natural products of traditional medicine, which provided for many decades significant hints to discover important drugs, that are still in use in several human diseases (for review see Cragg et al., 2014 and Newman and Cragg, 2016). In search of novel anti-inflammatory molecules to reduce the adverse effects of chronic lung inflammation in CF patients, we were inspired by serendipity on the potential activities of black seeds collected from the deserts of Northern Africa (Morocco) and distributed by Berber pharmacists (Supplementary Text S1). However, safety issues referred to the straight use of extracts from natural products have been raised (Ekor, 2014), which prompted us to identify and test the major single chemical compounds extracted from this natural product.

We focused here on extract from the black seeds of *N. arvensis*. Few data have been reported on “*N. arvensis,”* e.g., the online PubMed search only recalls seven articles, suggesting mainly generic antimicrobial and anti-inflammatory activities (Landa et al., 2009). Here we found that the extract of *N. arvensis* has anti-inflammatory activity in bronchial epithelial cells exposed to the Gram-negative bacterium *P. aeruginosa* (**Figures 2, 3**), a classical model system to test relevant molecules for CF lung inflammation. We excluded an artifactual effect on cell viability (**Figure 2E**) and, being the chloroform extract of *N. arvensis* active on Gram-positive bacteria (Landa et al., 2009), we also excluded that the anti-inflammatory effect could be mediated by an anti-bacterial activity or by inhibition of bacterium binding to the bronchial epithelial cells (**Figures 2C,D**). We focused on the three most abundant components extracted with chloroform, which were validated with their pure standards (**Table 1**), namely BSS, stigmasterol, and campesterol (**Figure 1**), which are the three most abundant sterols deriving from plants (Valitova et al., 2016). Since plant sterols have been widely tested for efficacy and safety in clinical pharmacology of highly relevant human diseases, such as dyslipidemia related to atherosclerosis, and since consensus has been reached on the absence of adverse signals in large scale clinical trials (Gylling et al., 2014), we felt justified in pursuing a repurposing strategy toward CF lung inflammation. Moreover, different mixtures of these plant sterols have been found of potential immunomodulatory effect (Bouic and Lamprecht, 1999; Bouic, 2002; Yuk et al., 2007).

We found that BSS (100 nM) significantly reduced the expression of the major neutrophilic chemokines IL-8, GRO-α, and GRO-β in human bronchial epithelial cells challenged with *P. aeruginosa*. This is consistent with a seminal observation showing that BSS partly reduces IL-8 in skin fibroblasts challenged with retinoic acid (Kim et al., 2003). How could we possibly explain the effect of BSS on neutrophilic chemotaxis? A plant-derived sterol mixture including BSS was found to inhibit the recruitment of neutrophils in a murine model of carrageenan-induced inflammation *in vivo* (Navarro et al., 2001). The effects of BSS on pro-inflammatory signal transduction have not been extensively investigated, the only hint being that BSS was found to inhibit the active form of the PKCα in prostate-derived cells (Kassen et al., 2000). Here we found that BSS partly reduces the activation of the PKCα isoform in a completely different model, namely the bronchial epithelial cells challenged with *P. aeruginosa* (**Figure 7**), which we found involved with a relevant pro-inflammatory role in the Ca^2+^-dependent signaling machinery, leading to IL-8 gene expression induced by *P. aeruginosa* in CF bronchial epithelial cells (Bezzerri et al., 2011b). The role of BSS in the organization of lipid rafts of plasma membranes and in the modulation of ceramide (Beck et al., 2007; Hąc-Wydro, 2013; Grosjean et al., 2015) also opens interesting insights in relation to sphingolipid metabolism and inflammation in CF lung disease (for review see Aureli et al., 2016).

Taken together, these results suggest that pharmaceutically relevant concentrations of BSS are promising in down-modulating the key neutrophil chemokines strongly induced in bronchial epithelial cells derived from CF patients upon response to *P. aeruginosa in vitro* (Bezzerri et al., 2011a; Prandini et al., 2016). The large use of BSS in controlled clinical trials, presenting a satisfactory safety profile for long term use (Gylling et al., 2014), stimulates to investigate further on the mechanism(s) of action of this molecule in the CF respiratory models, both *in vitro* and *in vivo*, for potential application as an anti-inflammatory molecule complementary to CFTR gene-directed modulators.

## Author Contributions

Conception: MA, MD, PP, GS, RG, and GC. Design: IL, MD, AR, GS, RG, and GC. Acquisition: IL, VB, ED, EN, AG, MT, SM, and AS. Analysis and interpretation: IL, MD, AG, AT, GL, GS, RG, and GC. Drafting the manuscript for important intellectual content: IL, MD, GL, GS, RG, MA, and GC.

## Conflict of Interest Statement

The authors declare that the research was conducted in the absence of any commercial or financial relationships that could be construed as a potential conflict of interest.
