# Supplementary material for: β-Sitosterol Reduces the Expression of Chemotactic Cytokine Genes in Cystic Fibrosis Bronchial Epithelial Cells
Source: Front Pharmacol. 2017 May 12;8:236. doi: 10.3389/fphar.2017.00236 (PMC5427149; doi:10.3389/fphar.2017.00236)
Supplement: Supplementary file 1 [file Data_Sheet_1.DOCX]

Supplementary Material

β-Sitosterol Reduces the Expression of Chemotactic Cytokine Genes in Cystic Fibrosis Bronchial Epithelial Cells

**Supplementary Text S1**

A 8-year-old female child originating from Morocco, living with her family in Verona, Italy, presented to the pediatric clinic of the co-author M.A. in Verona with recurrent nocturnal moderate bronchial asthma acute attacks, wheezing in inter-critical periods and in basal conditions, shortness of breath, most often precipitated by viral respiratory infections and exercise. Allergy skin testing was positive for both grasses and house dust mite *D. pteronyssinus*. Lung function testing confirmed reduced values of vital capacity, forced expiratory volume (FEV_1_) and FEF_25-75_ flow rates, which tended to normalize following administration of aerosolized broncho-dilators. She received a diagnosis of bronchial asthma and was treated with inhaled corticosteroid (fluticasone propionate 100 mcg) associated with broncho-dilator (salmeterol 50 mcg) twice a day during all the year (March 2010). This regimen was providing poor remission of the symptoms, requiring additional weekly periods with oral corticosteroids (prednisone 25 mg in decreasing doses over 5-7 days ) during the asthma attacks. Then, the child followed her family back to Morocco for Christmas holidays (December 2011), missing pediatric visits for about 8 months. At the first summer follow up visit at the end of this period (August 2012), her mother reported that, during her stay in Morocco, the drugs prescribed by the Italian pediatrician finished. Thus the child was treated only with "black seeds of the desert" purchased at the local Berber's pharmacy near Marrakech. The black seeds were ground, mixed with honey, stored in a glass jar and given to the child (one teaspoon twice a day). The symptoms were reported by her mother to be under good control and the black seed remedy was prolonged for months even when the family was again back to Italy. The first follow-up visit to the pediatric clinic confirmed that the child in treatment with the black seeds was in good clinical conditions: no more asthma attacks, wheezing nor shortness of breath (August 2012). One month later, the black seeds stock finished, the symptoms reappeared and the second follow-up visit in the pediatric clinic confirmed the recurrence of the symptoms in the absence of any treatment, thus requiring the association of prednisone propionate and salmeterol to keep symptoms under control (November 2012). The clinical corse reported here suggested the pediatrician to investigate the potential anti-inflammatory properties of the black seeds utilized by the Moroccan family for the child affected by chronic respiratory inflammation. The parents and the child were given informed consent and their approval was obtained.

Child's mother was invited to provide the pediatrician a sample of the same black seeds utilized and purchased from the Berber's pharmacy near Marrakech. Black seeds from the same source were then obtained from Morocco and provided to the investigators. A fraction of the seeds were sowed in small pots for germination and stored in greenhouses. The seedlengs were then transferred to an open field of the Botanical Garden of the University of Ferrara, Italy until the flowering (February 2013). Adult flowering plants were authenticated by the Curator of the Botanical Garden Dr. Fabrizio Negrini as belonging to the species *Nigella arvensis*. The majority of the black seeds remaining were then treated to extract the chemical compounds as described under Material and Method.
